# Adverse Effects of Meditation: Autonomic Nervous System Activation and Individual Nauseous Responses During Samadhi Meditation in the Czech Republic

**DOI:** 10.1007/s10943-024-02024-5

**Published:** 2024-04-12

**Authors:** Silvie Kotherová, Jakub Cigán, Lenka Štěpánková, Mária Vyskočilová, Simona Littnerová, Anastasia Ejova, Milan Sepši

**Affiliations:** 1 Department of Sociology, Andragogy and Cultural Anthropology, Palacký University Olomouc, Olomouc, Czech Republic; 2 Laboratory for the Experimental Research of Religion (LEVYNA), Department for the Study of Religions, Faculty of Arts, Masaryk University, Brno, Czech Republic; 3 Department of Psychology, Faculty of Social Studies, Masaryk University, Brno, Czech Republic; 4 Psychology Research Institute–Research departments, Faculty of Social Studies, Masaryk University, Brno, Czech Republic; 5 Department of Internal Medicine and Cardiology, University Hospital Brno, Brno, Czech Republic; 6 Department of Internal Medicine and Cardiology, Faculty of Medicine, Masaryk University Brno, Brno, Czech Republic; 7 Institute of Biostatistics and Analyses, Faculty of Medicine, Masaryk University, Brno, Czech Republic; 8 School of Psychology, Faculty of Health and Medical Sciences, The University of Adelaide, Adelaide, Australia; 9 School of Psychological Sciences, Faculty of Medicine, Health and Human Sciences, Macquarie University, Sydney, Australia

**Keywords:** Meditation, Samadhi, Nausea, Autonomic nervous system, Heart rate variability, Vasovagal syncope

## Abstract

Buddhist meditation practices, including Samadhi meditation, which forms the basis for mindfulness practice, are broadly promoted as pathways to wellbeing, but evidence of their adverse effects is emerging. In a single-group observational study with assessments of autonomic system before, during, and after Samadhi meditation, we explore the relationship between post-meditation nausea symptoms and the degree of change in autonomic system activity during meditation as compared to before and after in 57 university students (42 women; mean age = 22.6) without any previous experience in meditation or yoga practices. We hypothesize that nauseous feelings in meditation are connected to a rapid increase of activity in the sympathetic nervous system, as indicated by decreased heart-rate variability (HRV). We additionally explore links between meditation-induced nausea and two markers of parasympathetic activity: increased HRV and vasovagal syncope. Engaging in meditation and increased nausea during meditation were both associated with increased markers of HRV parasympathetic activity, but 12 individuals with markedly higher nausea demonstrated increased HRV markers of sympathetic activity during meditation. Vasovagal syncope was observed but found to be unrelated to nausea levels. Drivers of adverse effects of meditation in some individuals require further investigation.

## Introduction

Meditation is the process of training attention and awareness to achieve a physiological state that elicits physical and mental relaxation and enhances emotional stability (Bajaj et al., 2019; Jevning et al., 1992; Lee et al., 2015; Young & Taylor, 1998). Together with the associated state of mindfulness—being non-judgmentally focused wholly on the present moment—meditation practice has been found to have positive effects on mental and physical health (Arias et al., 2006; Grossman et al., 2004; Hofmann et al., 2010; Lutkajtis, 2019a). With respect to mental health, positive effects have been observed mainly for reducing symptoms of stress and depression, although benefits for anxiety have been observed when meditation or mindfulness are practiced in conjunction with other treatment approaches (Saeed et al., 2019). Observed physical benefits include improved sleep quality (Rusch et al., 2019), and reduced blood pressure and heart rate, implying reduced risk of cardiovascular disease (Conversano et al., 2021; Kayloni & Emery, 2015; Krittanawong et al., 2020; Levine et. al, 2017).

Reviewers have consistently called for increased theoretical and methodological rigor when exploring mechanisms underlying the effects of meditation (Hickey, 2010; Lomas et al., 2015; Ospina et al., 2007; Younge et al., 2015), especially with respect to cardiovascular functioning, where meta-analyses of the direction of effect have shown mixed results (Conversano et al., 2021; Dawson et al., 2020; Olex et al., 2013; Schnaubelt et al., 2019; Scott-Sheldon et al., 2020; Zhang et al., 2021). Proposals regarding mechanisms linking meditation and cardiovascular functioning focus on stress reduction. Stress is associated with continued activation of the sympathetic (‘fight-or-flight’) nervous system without counteraction by the parasympathetic (‘rest-and-digest’) nervous system. Long-term stress results in hormonal imbalances and maladaptive lifestyle patterns (e.g., alcohol use, lack of exercise, and poor sleep) that represent risk factors for hypertension and cardiovascular disease (Conversano et al., 2021; Won & Kim, 2016). Arguably, meditation can reduce sympathetic nervous system hyperactivation because it increases parasympathetic activity while reducing sympathetic activity or leaving it unchanged (Gerritsen & Band, 2018; Kozhevnikov, 2019; McCorry, 2007; Shr-Da & Pei-Chen, 2008). However, in what is known as the ‘meditation paradox’, it has been observed that, in some individuals, certain relaxation and meditation techniques produce increased heart rate—an indication of sympathetic activation (Peng et al., 2004). As highlighted in a narrative review, neuroimaging patterns and subjective reports also suggest increases in general arousal and alertness following some forms of meditation (Britton et al., 2014).

In suggesting that some people might not experience parasympathetic dominance during meditation, the meditation paradox is consistent with the observation by some reviewers that theoretical perspectives on the health benefits of meditation lack clarity with respect to possible adverse effects of meditation practice (Lauricella, 2014). A recent narrative critique of literature on the health benefits of meditation emphasizes that only a small number of studies have explored adverse effects, largely due to a broader motivation in the media and the scientific community to present meditation as a ‘simple’ side-effect-free solution to various health problems (Lutkajtis, 2019a). Similarly, systematic reviews indicate that few trials of mindfulness-based therapies track adverse effects (Wong et al., 2018), partly because there are currently no requirements to report them or guidelines for doing so (Farias et al., 2020).

A documented negative side-effect of one type of meditation, Ānāpāna smrti, is nausea: a complex reaction of different physiological and psychological systems manifesting as epigastric discomfort combined with an urge to vomit and associated with increased sympathetic activity (LaCount et al., 2011; Muth et al., 1996; Sclocco et al., 2016). This side-effect has been discussed in ancient and contemporary meditation manuals, as well as qualitative reports from experienced meditators (Lindahl et al., 2017). In our own study of Ānāpāna smrti meditation, 17% of participants reported dizziness, nausea, extreme sweating, hot flushes, weakness, and faintness during meditation (Kotherová, 2015).

In the present study, we investigate whether feelings of nausea during Ānāpāna smrti meditation reflect increased sympathetic activity. The reverse pattern—an association between nausea and increased *parasympathetic* activity—is also possible, given that nausea often accompanies vasovagal syncope, a benign temporary decrease in blood flow to the brain caused by parasympathetic activation and observed during meditation (Alboni, 2015; Goshvarpour & Goshvarpour, 2021).

Our protocol involves measuring heart-rate-variability (HRV) indices of sympathetic and parasympathetic activation, as well as blood flow indicators, during three phases: (1) before, (2) during, and (3) after Ānāpāna smrti meditation. Feelings of nausea were assessed at the end of the third phase (i.e., post-meditation), and the analysis focuses on analyzing associations between the degree of reported nausea and the degree of change in heart-rate and blood flow parameters as participants moved from a resting state (pre-meditation) to the meditation phase and to rest again (post-meditation).

One notable strength of our protocol is that we recruit participants without prior meditation experience. Past studies of autonomic nervous system activity during meditation have tended to focus on experienced practitioners (Britton et al., 2014; Gerritsen & Band, 2018; Peng et al., 2004).

A further strength is that our choice of meditation technique is theoretically motivated. This is critical, given that different philosophical traditions, ritual practices and techniques for meditation have been shown to produce different physiological and behavioural effects (Brook et al., 2013; Kozhevnikov, 2019; Krygier et al., 2013; Lehrer et al., 1999; Levine et al., 2017; Lutz et al., 2008; Raffone et al., 2019; Rubia, 2009; Tang et al., 2009). We explore the effects of a meditation technique known to heighten parasympathetic activity (Kozhevnikov, 2019; Ñāṇamoli, 1998). Ānāpāna smrti (san.), also known as Ānāpāna sati (pál.), belongs to a group of Theravada Buddhist meditation techniques commonly known as Samadhi or Shamatha (Ñāṇamoli, 1998). Samadhi practice involves training concentration, and so practitioners are instructed to direct their “undivided attention” to the single object of meditation while withdrawing their focus from other objects (Buddhaghosa, 2010). Ānāpāna smrti practice is usually translated as “breath (ānāpāna) awareness (smrti)”. Instructions specifically concern mindful observation of breath inhalation and exhalation. During the meditation, the person focuses their mind on a singular object or process with eyes closed and trying to be aware as much as possible of internal bodily processes.

Overall, in investigating autonomic nervous system activity potentially responsible for nausea during Samadhi meditation, we hypothesize that higher levels of nausea will be associated with higher levels of activity in the sympathetic arm of the autonomic nervous system during meditation as compared to rest. Autonomic nervous system activity will be measured using HRV indices, but blood pressure (blood flow) will also be monitored during meditation and rest to explore known associations between nausea and increased parasympathetic activity, as reflected in increased probability of vasovagal syncope.

## Method

### Participants

Fifty-seven undergraduate students enrolled in an undergraduate university-wide research methods course (42 women; mean age = 22.6; SD = 1.7) participated in the study in exchange for course credit. All students enrolled in the course (approximately 150) had to complete a preliminary screener survey, and the participants in our study were invited to participate based on their screener survey responses. To be eligible for the study, students had to report no prior experience with any kind of meditation or yoga practice. Participants also had to report absence of a current mental health disorder, chronic heart condition or history of psychoactive drug use. For more detailed information about the participants, see Table 1 in the Results.

**Table 1:** Preliminary descriptive analysis comparing nausea groups

	Low nausea (*n* = 45)	High nausea (*n* = 12)	Significance of group differences^
Age	22.96 (1.99)	22.25 (1.55)	Means: *p* = .33 SDs: *p* = .59
Gender	W: 30 M: 15	W: 12 M: 0	Proportions: *p* = .02*
Meditation–demanding	3.40 (1.71)	4.67 (1.88)	Means: *p* = .04* SDs: *p* = .74
Meditation–uncomfortable	1.42 (1.71)	3.91 (4.09)	Means: *p* = .01** SDs: *p* = .07
Meditation–success	5.84 (0.64)	5.50 (1.24)	Means: *p* = .40 SDs: *p* = .002**
Meditation–errors	3.11 (3.37)	4.42 (5.07)	Means: *p* = .03* SDs: *p* = .08
Meditation–max. number	73.09 (47.79)	64.25 (47.61)	Means: *p* = .001*** SDs: *p* = .70
DC at T1	11.47 (5.12)	10.59 (2.76)	Means: *p* = .98 SDs: *p* = .15
LF at T1	66.75 (17.05)	68.23 (13.98)	Means: *p* = .94 SDs: *p* = .70
HF at T1	33.25 (17.05)	31.77 (13.98)	Means: *p* = .94 SDs: *p* = .70
LF/HF ratio at T1	2.85 (2.02)	3.06 (2.37)	Means: *p* = .94 SDs: *p* = .64

^Fisher’s exact test for Gender; Poisson regression for counting errors and max. number; otherwise Wilcoxon sum-rank test for means, and Levene’s test for SDs: * .01 < *p* ≤ .05, ** .001 < *p* ≤ .01, *** *p* ≤ .001

### Procedure

All participants provided written informed consent to participate in a study investigating physiological changes associated with cognitive load in a mental exercise. The word “meditation” was not mentioned at any point during recruitment and data collection to avoid creating expectations around what the effect of mental exercise should be (Lustyk et al., 2009).

The experiment took place at the Department of Internal Medicine and Cardiology at **XY** University Hospital. Participants were asked (a) not to consume alcoholic beverages, drugs or energy drinks for at least 24 h before taking part in the study, (b) not to consume caffeinated beverages for at least six hours before, and (c) refrain from eating for at least 2 h and follow a sufficient drinking regime up to 3 h before. During the entire procedure, participants were under supervision of a doctor and nurse. Testing took place in a quiet private room.

After being fitted with monitoring devices (see Measures: Equipment below), each participant underwent three phases in a set order: the pre-meditation phase (Phase 1; T1), the meditation phase (Phase 2; T2) and the post-meditation phase (Phase 3; T3). During the pre-meditation phase, the participant was instructed to sit quietly with their eyes open for five minutes while all heart-rate measures were collected. During the meditation phase, the same measures were collected during a 10-min meditation exercise described in the ‘Meditation’ section below. The post-meditation phase was identical to the pre-meditation phase.

### Meditation

At the beginning of the meditation phase, participants were provided written instructions to sit quietly with their eyes closed and focus on their breathing by counting when breathing in and breathing out. Participants were further instructed to begin counting from the beginning if they found their attention being drawn away. The instructions were as follows:

When prompted, please close your eyes and keep them closed until told otherwise. Focus your attention on your breath. Focus on your inhalation and exhalation. While focusing on your breath, begin to count in your mind as follows: on inhalation say 1, on exhalation say 1. On the next inhalation say 2, and on the next exhalation say 2. Continue like this: 3–3, 4–4, and so on. Try not to lose your focus on the breath. If you lose focus (e.g., if you start following another thought or focus on some discomfort or emotion), start counting from the beginning; so, again, from the number 1 (1–1, 2–2 etc.). If you are unsure about any aspect of these instructions, feel free to ask the research assistant.

Participants were further informed that the task was not a race to the highest breath count, but an exercise in being present with the breath as much as possible. That is, they were informed that the goal of the task was not to reach the highest count, but to focus on breathing, seeing the counting as an aid to that process. Participants performed the meditation task for 10 min.

### Measures

#### Equipment

A continual ECG Holter monitoring device (MARS 5000, GE Marquette Medical Systems, Milwaukee, United States) was used for measuring heart rate. A Portapress Model 2 (Finapres Medical System B.V. Amsterdam, The Netherlands), was used for non-invasive continuous blood pressure measurement. Additionally, outside the scope of the present study, a patient monitor (Dash 4000, GE Healthcare, Illinois, United States) was used for monitoring of breathing rate and blood oxygen saturation.

#### Heart Rate Variability Indices of Autonomic Nervous System Activity

Among the HRV measures, the Deceleration capacity (DC) of the heart rate served as an indicator of parasympathetic activity (Bauer et al., 2006). Normalized power in the low-frequency range (LF; 0.04–0.15 Hz; Malik, 1996) served as a marker of sympathetic activity, while normalized power in the high-frequency range (HF; 0.15–0.4 Hz; Malik, 1996) served as an additional marker of parasympathetic activity. The ratio of LF to HF (LF/HF) was calculated as a marker of sympathetic system dominance (i.e., of whether any sympathetic activation is larger in magnitude than parasympathetic activation; Kiyono et al., 2017).

#### Blood-Pressure Indices of Parasympathetic Nervous System Activity (Vasovagal Syncope)

Instances of vasovagal syncope in Phases 2 (meditation) and 3 (post-meditation) were identified by two independent cardiologists (the fourth and last authors, blind to participant allocation to nausea groups), who discussed conflicting judgements and came to a consensus. To arrive at the judgments, the cardiologists viewed, for each participant in each phase, graphs of mean arterial pressure plotted concurrently with beats-per-minute (see Part 2 of the Online Supplement for the graphs and judgements; DeMers & Wachs, 2019). For each participant, presence of vasovagal syncope in Phase 2 (meditation) or Phase 3 (post-meditation) was, finally, operationalized as a binary variable: 0 (No) or 1 (Yes).

#### Nausea Symptoms

Nausea symptoms were measured (at the end of Phase 3: post-meditation) using a Nausea Profile (Muth et al., 1996), a measure in which participants were instructed to indicate the extent to which they were feeling each of the following 17 symptoms on a scale from of 0 (not at all) to 10 (severely): “shaky”, “upset”, “lightheaded”, “sick”, “sweaty”, “queasy”, “worried”, “hopeless”, “fatigued/tired”, “panicked”, “nervous”, “scared/afraid”, “ill”, “discomfort in my stomach”, “might vomit”, “weak”, and “hot/warm”. In a back-translation process, the Nausea Profile was translated from English to Czech and then back to English by two independent translators and the final translation was compared to the original by a third person. The final nausea score was the sum of all responses, expressed as a percentage of the maximum possible score (170), in line with Muth et al. (1996)’s specifications. Thus, scores could range from 0 to 100, and reflected a well-established multifaceted conceptualisation of nausea as a composite of three correlated latent factors: somatic distress, gastrointestinal distress, and emotional distress (Bedree et al., 2023; Sanger & Andrews, 2023; Tarbell et al., 2023).

#### Additional Measures: Experiences During Meditation

At the end of Phase 3 (the post-meditation phase), participants were asked about different aspects of their meditation experience, including the extent to which they found the task demanding (“Please indicate how difficult you find the breath counting exercise:___” -3 = not at all, 3 = extremely). Participants were also asked about the extent to which they felt uncomfortable during the task (“To what extent during the breath counting exercise did you feel uncomfortable?” -3 = not at all, 3 = extremely), and the extent to which they felt they were successful at the task (“Please indicate how successful you think your breath-counting practice was:__”, -3 = not at all, 3 = extremely). Participants were also asked to estimate the number of counting errors they made during the task (“Try to estimate how many times you had to go back to count: __”), and to report the maximum number of breaths they were able to count during the task (“What is the highest number you were able to count to without interruption? __”).

To enable tracking of study artifacts, participants completed a final open-ended question, in which they were asked to describe what they experienced during the meditation (“Describe as accurately as possible how you experienced the experiment”). No participants were excluded on the basis of this description.

### Statistical Analysis

**Fig. 1:**
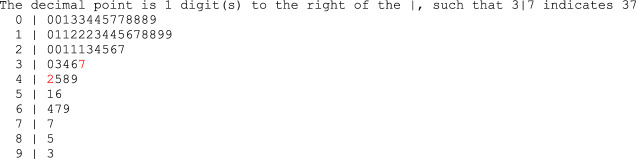
Stem-and-leaf plot of nausea scores, with the cut-offs for the identified nausea groups shaded in red

**Fig. 2:**
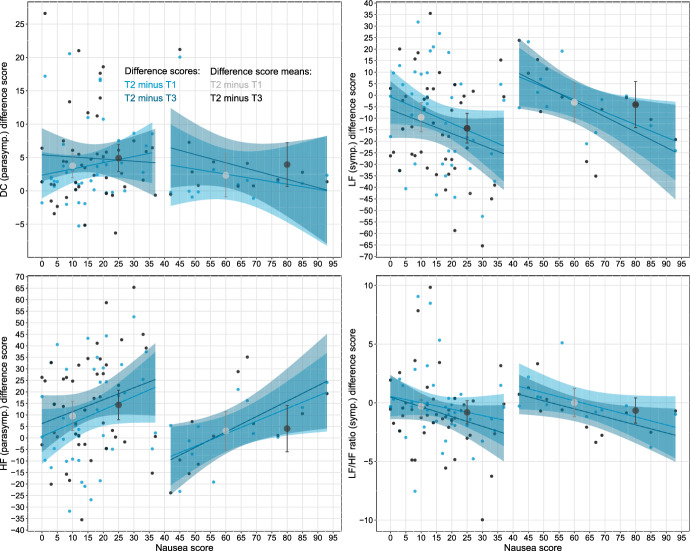
Descriptive statistics for HRV difference scores–scatter plots, means (larger dots), and slopes indicating correlation strength

To address the main research question regarding the relationship between HRV indices of autonomic (sympathetic and parasympathetic) activity and nausea, we fitted eight mixed effects models – two for each HRV outcome of interest: DC, LF, HF and the LF/HF ratio. (Fig. 1) The models were informed by the fact that we observed two groupings of nausea scores—low and high (see Results: Descriptive statistics). In the models, the fixed effects were phase/timepoint (two levels: Phase 2 meditation vs. Phase 1 pre-meditation in one model, and Phase 2 meditation vs. Phase 3 post-meditation in the other model), nausea symptoms, nausea group (two levels: high vs. low), and all possible two-way interactions. The random effect was participant ID. Effectively, the two models for each outcome differed only in terms of the phases/timepoints they compared. Models were fitted using the *lme4* package in *R* v. 4.2.3 (Bates et al., 2014). Descriptive graphs capturing phases/timepoints as difference scores (T2 minus T1 and T2 minus T3; i.e., Phase 2 minus Phase 1 and Phase 2 minus Phase 3) were drawn to assist with interpretation (see Fig. 2). Sample analysis code is available in Part 1 of the Online Supplement. Two model coefficients were of interest, given the research question: the timepoint x nausea symptom score interaction (indicating whether the degree of HRV change from rest to meditation would change depending on nausea score), and the timepoint by group interaction (indicating whether the degree of HRV change from rest to meditation would change depending on whether a participant’s nausea symptom score was, at a coarse-grained level, low or high).

To examine associations between nausea and increased parasympathetic activity, as reflected in increased probability of vasovagal syncope, we fitted a logistic regression in which presence (yes/no) of vasovagal syncope in Phases 2 (meditation) or 3 (post-meditation) was the outcome variable, and the predictors were nausea group, nausea symptom score, and an interaction term. Thus, we allowed for the possibility that the strength of the association between nausea symptoms and probability of syncope would differ by nausea group.

The characteristics of the two nausea groups on HRV indices at baseline and additional available measures of meditation experience were explored in a preliminary descriptive analysis involving independent-samples *t*-tests and Chi-square tests (see Results: Descriptive statistics).

## Results

### Descriptive Statistics

As shown in Fig. 1—a stem-and-leaf plot of nausea scores—we found that nausea symptom scores formed two clusters, in that no scores fell between 37 and 42. Based on this finding and the descriptive scatter plots in Fig. 2, we modelled the HRV-nausea relationship separately for the two clusters. In a finding that provides support for this decision, Muth et al. (1996) found 42 to be the average nausea level experienced during optokinetic rotation by people prone to motion sickness. Table 1 summarises how the clusters compared with respect to demographics, meditation experience, and baseline HRV indices. High scorers (*n* = 12) differed from low scorers (*n* = 45) in that they were all women, more likely to find meditation demanding and uncomfortable, more likely to make errors during meditation, and likely to count to a lower number during meditation.

### The HRV-Nausea Relationship

The results of the mixed-effects modelling assessing the relationship between nausea symptoms and meditation-related changes in each of the HRV indices – DC and HF (markers of parasympathetic activity) and LF and the LF/HF ratio (markers of sympathetic activity) – are presented in Table 2. Figure 2 plots the associated descriptive statistics. Results indicate that, in line with our hypothesis, higher nausea, in terms of group membership, was associated with increased sympathetic activity, in that the timepoint x group interaction effect was significant and positive for both models for LF and the second model (of the last two timepoints: T2 and T3) for the LF/HF ratio. In line with the previously observed relationship between higher nausea and vasovagal syncope, a marker of parasympathetic activity, we also observed a positive correlation between HF—a marker of parasympathetic activity – and nausea in both associated models (for T2 and T1, as well as T2 and T3). Notably, across both groups, and models for both the first two timepoints and last two, we observed non-zero change levels in DC (i.e., a main effect of timepoint), indicating an increase in parasympathetic activity during meditation.

**Table 2:** Results and interpretation of the mixed effects modelling

Coeff.	Value (SE)	Sig.^	Mapping to Fig. 2 and interpretation
*DC—a marker of parasympathetic activity*			
Model for first two timepoints: intercept	12.21 (1.53)	< .001***	The central tendency of light blue dots (T2 minus T1), represented by the large light grey dot, is significantly greater than 0. Implies increased parasympathetic activity during meditation
Timepoint (T2(1) vs. T1(0))	2.21 (1.00)	.03*	
Nausea score	0.04 (0.08)	.68	
Group (high(1) vs. low(0))	3.98 (6.52)	.54	
Timepoint (T2(1) vs. T1(0)) x nausea score	0.01 (0.05)	.81	
Timepoint (T2(1) vs. T1(0)) x group (high(1) vs. low(0))	− 1.33 (2.76)	.63	
Nausea score x group	− 0.11(0.13)	.41	
Model for last two timepoints: intercept	11.05 (1.18)	< .001***	The central tendency of dark blue dots (T2 minus T3), represented by the large dark grey dot, is significantly greater than 0. Implies increased parasympathetic activity during meditation
Timepoint (T2(1) vs. T3(0))	3.69 (1.17)	.002**	
Nausea score	0.08 (0.06)	.24	
Group (high(1) vs. low(0))	2.82 (5.02)	.57	
Timepoint (T2(1) vs. T3(0)) x nausea score	− 0.03 (0.06)	.55	
Timepoint (T2(1) vs. T3(0)) x group (high(1) vs. low(0))	1.26 (3.15)	.69	
Nausea score x group	− 0.12 (0.10)	.22	
*LF—a marker of sympathetic activity*			
Model for first two timepoints: Intercept	61.87 (4.70)	< .001***	The light blue line slopes are significantly negative; i.e., the light blue dots (T2 minus T1) are more likely to be smaller/negative for people with higher nausea scores, implying decreased sympathetic activity during meditation with increasing nausea
Timepoint (T2(1) vs. T1(0))	− 0.17 (3.13)	.96	
Nausea score	0.07 (0.26)	.78	
Group (high(1) vs. low(0))	− 2.23 (20.07)	.91	
Timepoint (T2(1) vs. T1(0)) x nausea score	− 0.40 (0.16)	.01**	
Timepoint (T2(1) vs. T1(0)) x group (high(1) vs. low(0))	22.76 (8.46)	.01**	The central tendency of light blue dots (T2 minus T1), represented by the large light grey dot, is significantly higher in the High Nausea group, implying that this group is more likely to experience increased and unchanged (rather than reduced) sympathetic activity during meditation
Nausea score x group	0.04 (0.40)	.92	
Model for last two timepoints: intercept	64.51 (4.34)	< .001***	As for slopes and central tendency in model above, but applied to the dark blue and dark grey dots, as well as the dark blue slope lines (T2 minus T3)
Timepoint (T2(1) vs. T3(0))	− 3.24 (3.30)	.33	
Nausea score	0.06 (0.24)	.80	
Group (high(1) vs. low(0))	− 8.16 (18.51)	.66	
Timepoint (T2(1) vs. T3(0)) x nausea score	− 0.42 (0.16)	.01**	
Timepoint (T2(1) vs. T3(0)) x group (high(1) vs. low(0))	26.70 (8.87)	.01**	
Nausea score x group	0.11 (0.37)	.76	
*HF—a marker of parasympathetic activity*			
Model for first two timepoints: intercept	38.13 (4.70)	< .001***	The light blue line slopes are significantly positive; i.e., the light blue dots (T2 minus T1) are more likely to be larger/positive for people with higher nausea scores, implying increased parasympathetic activity during mediation with increasing nausea
Timepoint (T2(1) vs. T1(0))	0.17 (3.13)	.96	
Nausea score	− 0.07 (0.26)	.78	
Group (high(1) vs. low(0))	2.23 (20.07)	.91	
Timepoint (T2(1) vs. T1(0)) x nausea score	.40 (0.16)	.01**	
Timepoint (T2(1) vs. T1(0)) x group (high(1) vs. Low(0))	− 22.76 (0.40)	.01**	The central tendency of light blue dots (T2 minus T1), represented by the light grey dot, is significantly lower in the High Nausea group, implying that this group is more likely to experience decreased and unchanged (rather than increased) parasympathetic activity during meditation
Nausea score x group	− 0.04 (0.40)	.92	
Model for last two timepoints: intercept	35.49 (4.34)	< .001***	As for slopes and central tendency in model above, but applied to the dark blue and dark grey dots, as well as the dark blue slope lines (T2 minus T3)
Timepoint (T2(1) vs. T3(0))	3.24 (3.30)	.33	
Nausea score	− 0.06 (0.24)	.80	
Group (high(1) vs. low(0))	8.16 (18.51)	.66	
Timepoint (T2(1) vs. T3(0)) x nausea score	0.42 (0.16)	.01**	
Timepoint (T2(1) vs. T3(0)) x group (high(1) vs. low(0))	− 26.70 (8.87)	.002**	
Nausea score x group	− 0.11 (0.37)	.76	
*LF/HF ratio—a marker of sympathetic activity dominance*			
Model for first two timepoints: intercept	3.22 (0.56)	< .001***	
Timepoint (T2(1) vs. T1(0))	0.56 (0.46)	.22	
Nausea score	− 0.02 (0.03)	.42	
Group (high(1) vs. low(0))	− 2.09 (2.39)	.38	
Timepoint (T2(1) vs. T1(0)) x nausea score	− 0.04 (0.02)	.06	
Timepoint (T2(1) vs. T1(0)) x group (high(1) vs. low(0))	1.24 (1.74)	.08	
Nausea score x group	0.06 (0.05)	.24	
Model for last two timepoints: intercept	3.23 (0.56)	< .001***	The dark blue line slopes are significantly negative; i.e., the dark blue dots (T2-T3) are more likely to be smaller/negative for people with higher nausea scores, implying decreased sympathetic activity during meditation with increasing nausea
Timepoint (T2(1) vs. T3(0))	0.38 (0.50)	.44	
Nausea score	− 0.01 (0.03)	.80	
Group (high(1) vs. low(0))	− 1.76 (2.40)	.47	
Timepoint (T2(1) vs. T3(0)) x nausea score	− .06 (0.02)	.02*	
Timepoint (T2(1) vs. T3(0)) x group (high(1) vs. low(0))	2.71 (1.33)	.04*	The central tendency of the dark blue dots (T2 minus T3), represented by the large dark grey dot, is significantly higher in the High Nausea group, implying that this group is more likely to experience increased and unchanged (rather than reduced) sympathetic activity during meditation
Nausea score x group	0.04 (0.05)	.42	

^Type III Wald Chi-square test: * .01 < *p* ≤ .05, ** .001 < *p* ≤ .01, *** *p* ≤ .001

### The Syncope-Nausea Relationship

To directly assess the relationship between higher nausea and the probability of vasovagal syncope, we conducted a logistic regression in which presence of vasovagal syncope in Phase 2 (meditation) or 3 (post-meditation) was the outcome variable, and the predictors consisted of nausea symptom score, nausea group, and the group x score interaction. In the low nausea group, 20 participants (48%) were identified as having experienced vasovagal syncope at least once across Phases 2 and 3. Thus, in terms of descriptive statistics, results did not support the hypothesis, since syncope was less (rather than more) likely in the high nausea group. In the logistic regression, as Table 3 shows, neither group membership, nor nausea score, nor the combination of group membership and nausea score predicted the probability of syncope.

**Table 3:** Coefficients in the logistic regression of vasovagal syncope in T2 or T3 on nausea symptom score, nausea group, and the interaction term

Coeff	Value (SE)	Sig
Intercept	0.23 (0.59)	.70
Group (high(1) vs. low(0))	−4.82 (3.20)	.13
Nausea score	–0.02 (0.03)	.52
Nausea score x group	0.08 (0.06)	.15

## Discussion

Calls for theoretically guided investigations of cardiovascular functioning during various specific types of meditation have been made in light of observations of adverse effects of meditation in some individuals (e.g., Lauricella, 2014). The present study investigated heart rate variability and blood pressure (specifically, vasovagal syncope) in people practicing Samadhi meditation for the first time and reporting varying levels of the adverse side-effect of nausea. Overall, compared to a resting state, meditation was found to increase parasympathetic activity, although reports of high—compared to low—levels of nausea were associated with greater *sympathetic* activity during meditation. Meanwhile, in both the ‘low’ and ‘high’ nausea clusters, incremental increases in nausea were associated with incremental increases in parasympathetic activity. Further investigation involving blood pressure indicated that this association at the incremental level was not due to the effects of vasovagal syncope.

It might follow from our findings of correlated incremental increases in parasympathetic activity and nausea that, for people with an overactive sympathetic nervous system, Samadhi meditation—known for its activation of the parasympathetic nervous system—might produce a stronger parasympathetic response. To use a crude analogy, people who drive faster all the time (i.e., have an overactive sympathetic nervous system) also break harder all the time (i.e., are prone to stronger parasympathetic responses). We did not have the statistical power to investigate the effects of the interaction between nausea score and nausea group (i.e., cluster) on change in parasympathetic activity over time. A study powered to investigate such an interaction (through adding a three-way interaction to our mixed modelling framework) would be a logical next step.

Extending on dominant reviews in the area, our findings showed that activation of the sympathetic nervous system can be present not only in Vajrayana types of meditation (Kozhevnikov, 2019) but also in Samadhi meditation, which is the basic technique underlying mindfulness practice (Nilsson & Kazemi, 2016). Additionally, our study shows that, despite the parasympathetic system's general activation during Samadhi meditation, sympathetic nervous system activation and associated adverse physical symptoms can occur in some individuals (Farmer et al., 2015; Lutkajtis, 2019a;). This finding echoes Britton et al.’s (2014) observation in a narrative multidisciplinary review that meditation is a complex phenomenon that can elicit both parasympathetic and sympathetic nervous system activation—hypoarousal and hyperarousal—depending on dose, expertise, and individual factors.

On a practical level, our findings raise questions about whether meditation is ‘for everyone’. Our study identified adverse effects (nausea resembling motion sickness in terms of severity) in one out of five novice practitioners—12 out of 57 (21%). This is higher than the 11% rate of incidence of gastrointestinal symptoms reported in a recent systematic review of experimental, observational, and case-study-based evaluations of mindfulness practice (Farias et al., 2020).

People with high anxiety might stand to benefit most from the autonomic rebalancing meditation potentially offers, but, given the known proneness of more anxious people to nausea (e.g., Haug et al., 2002), longitudinal investigations are needed to determine whether people with high anxiety and stress levels tend to overcome the nausea barrier identified in the present study. Participants in the ‘high nausea’ group reported more challenges with meditation (i.e., greater demands and discomfort, and lower perceived success), so there is evidence that people are conscious of initial barriers, and could benefit from explicit support in overcoming them.

More broadly, our findings highlight that research into meditation and its utilization in healthcare should be based on a comprehensive understanding of the effects of various meditation techniques and their particular constituent elements. Despite pressure to characterize meditation as a low-cost intervention with widespread benefits, researchers must not lose sight of individual differences in physiological and psychological responding (Lutkajtis, 2019a, 2019b). Moreover, adverse effects should be documented when conducting trials of easy-to-access meditation and mindfulness apps (Taylor et al., 2022).

### Study Limitations

A key limitation of our findings is that we did not have a priori expectations of two nausea clusters, and thus had limited measures on which to compare and distinguish the ‘low’ and ‘high’ nausea groups. Clearer characterization of these groups is a task for future research. In light of recent findings identifying decreasing parasympathetic activity with increasing nausea in women but not men (Caillet et al., 2022), future research should seek to overcome the confounding effects of gender—effects that might have been present in the current study, given the gender imbalance across groups. Overall, we present findings that should inform future study designs and power analyses. We did not conduct a power analysis of our own, but our sample size exceeds or equals those of previous studies on adverse effects of meditation (Lindahl et al., 2017; Shapiro, 1992), and it was reassuring to see observed effects replicating across models in the ‘first two’ and ‘last two’ phases—models that differed only in terms of whether the baseline occurred before or after meditation.

A further limitation of our work is that we observed a positive relationship between nausea levels and HF, but not the other measured marker of parasympathetic activity—DC. Our findings are consistent with emerging evidence that the relationship between HF and DC is imperfect (Lewek et al., 2009; Pan et al., 2016), particularly at lower breathing rates, such as those likely induced by the meditation task in the present study (Wang et al., 2016). Future research should measure and report participants’ breathing rates.

Another potential limitation of our design is that, in exploring the relationship between meditation and parasympathetic activity, we focused on vasovagal syncope rather than smaller and more volatile changes in blood pressure and heart rate. Techniques for detecting these more fine-grained changes are emerging (van Dijk et al., 2020), and might allow for increased precision in capturing parasympathetic activity in future meditation research.

## Conclusions

In conclusion, we show that Samadhi meditation, which forms the basis for mindfulness practice, produces marked nausea symptoms in one-fifth of people attempting it for the first time. In line with findings that nausea can reflect both sympathetic (fight-flight) and parasympathetic (rest-digest) activation, we observed heightened heart-rate variability indices of sympathetic activation among the one-fifth of participants reporting stronger nausea, but also, in both groups, increasing HRV indices of parasympathetic activity with increasing reported nausea strength. We did not, however, observe increasing probabilities of vasovagal syncope (a blood-pressure-based index of parasympathetic activity) with increasing nausea levels. A future study would need to investigate whether some people (with heightened nausea and sympathetic activation) are prone to stronger parasympathetic (braking) responses. However, more broadly, our findings demonstrate how critical it is to monitor the potential negative side-effects of meditation, as well as the mechanisms contributing to them.

## Data Availability

Data sets generated and analyzed in the current study are available upon request from the corresponding author.

## References

[CR1] Alboni, P. (2015). The different clinical presentations of vasovagal syncope. *Heart,**101*(9), 674–678. 10.1136/heartjnl-2014-307096 25792719 10.1136/heartjnl-2014-307096

[CR3] Arias, A. J., Steinberg, K., Banga, A., & Trestman, R. L. (2006). Systematic review of the efficacy of meditation techniques as treatments for medical illness. *Journal of Alternative and Complementary Medicine (new York, N.y.),**12*(8), 817–832. 10.1089/acm.2006.12.817 17034289 10.1089/acm.2006.12.817

[CR4] Bajaj, B., Gupta, R., & Sengupta, S. (2019). Emotional stability and self-esteem as mediators between mindfulness and happiness. *Journal of Happiness Studies,**20*, 2211–2226. 10.1007/s10902-018-0046-4

[CR5] Bates, D., Mächler, M., Bolker, B., & Walker, S. (2014). Fitting linear mixed-effects models using lme4. *arXiv preprint *arXiv:1406.5823. http://arxiv.org/abs/1406.5823

[CR6] Bauer, A., Kantelhardt, J. W., Barthel, P., Schneider, R., Mäkikallio, T., Ulm, K., Hnatkova, K., Schömig, A., Huikuri, H., Bunde, A., Malik, M., & Schmidt, G. (2006). Deceleration capacity of heart rate as a predictor of mortality after myocardial infarction: Cohort study. *The Lancet,**367*, 1674–1681. 10.1016/S0140-6736(06)68735-7 10.1016/S0140-6736(06)68735-7 16714188

[CR7] Bedree, H., Tran, S. T., Koven, M. L., Wershil, S. J., Fortunato, J. E., & Essner, B. S. (2023). Impact of sleep disturbance on fatigue, nausea, and pain: mediating role of depressive symptoms among youth with disorders of gut-brain interaction. *Journal of Pediatric Gastroenterology and Nutrition,**77*(4), 468–473. 10.1097/mpg.0000000000003887 37434286 10.1097/MPG.0000000000003887

[CR9] Britton, W. B., Lindahl, J. R., Cahn, B. R., Davis, J. H., & Goldman, R. E. (2014). Awakening is not a metaphor: The effects of Buddhist meditation practices on basic wakefulness. *Annals of the New York Academy of Sciences,**1307*(1), 64–81. 10.1111/nyas.12279 24372471 10.1111/nyas.12279 PMC4054695

[CR10] Brook, R. D., Appel, L. J., Rubenfire, M., Ogedegbe, G., Bisognano, J. D., Elliott, W. J., Fuchs, F. D., Hughes, J. W., Lackland, D. T., Staffileno, B. A., Townsend, R. R., & Rajagopalan, S. (2013). Beyond medications and diet: Alternative approaches to lowering blood pressure: A scientific statement from the american heart association. *Hypertension,**61*, 1360–1383. 10.1161/HYP.0b013e318293645f 23608661 10.1161/HYP.0b013e318293645f

[CR11] Buddhaghosa. (2010). *Visuddhimagga: The path of purification* (4th ed.). Kandy: Buddhist Publication Society.

[CR12] Caillet, A. R., Russell, A. C., Wild, M. G., Acra, S., Bradshaw, L. A., Bruehl, S., & Stone, A. L. (2022). Sex moderates the relationship between Nausea severity and heart rate variability in adolescents and young adults. *Digestive Diseases and Sciences*. 10.1007/s10620-021-06892-9 33608817 10.1007/s10620-021-06892-9 PMC8373993

[CR14] Conversano, C., Orrù, G., Pozza, A., Miccoli, M., Ciacchini, R., Marchi, L., & Gemignani, A. (2021). Is mindfulness-based stress reduction effective for people with hypertension? A systematic review and meta-analysis of 30 years of evidence. *International Journal of Environmental Research and Public Health,**18*(6), 2882. 10.3390/ijerph18062882 33799828 10.3390/ijerph18062882 PMC8000213

[CR15] Dawson, A. F., Brown, W. W., Anderson, J., Datta, B., Donald, J. N., Hong, K., ... & Galante, J. (2020). Mindfulness‐based interventions for university students: A systematic review and meta‐analysis of randomised controlled trials. *Applied Psychology: Health and Well‐Being*, *12*(2), 384–410. 10.1111/aphw.12188 10.1111/aphw.12188 31743957

[CR17] DeMers, D., & Wachs, D. (2019). *Physiology, mean arterial pressure.* PMID: 30855814 30855814

[CR19] Farias, M., Maraldi, E., Wallenkampf, K. C., & Lucchetti, G. (2020). Adverse events in meditation practices and meditation-based therapies: A systematic review. *Acta Psychiatrica Scandinavica,**142*(5), 374–393. 10.1111/acps.13225 32820538 10.1111/acps.13225

[CR20] Farmer, A. D., Ban, V. F., Coen, S. J., Sanger, G. J., Barker, G. J., Gresty, M. A., Giampietro, V. P., Williams, S. C., Webb, D. L., Hellström, P. M., Andrews, P. L. R., & Aziz, Q. (2015). Visually induced nausea causes characteristic changes in cerebral, autonomic and endocrine function in humans. *The Journal of Physiology,**593*(5), 1183–1196. 10.1113/jphysiol.2014.284240 25557265 10.1113/jphysiol.2014.284240 PMC4358679

[CR22] Gerritsen, R. J., & Band, G. P. (2018). Breath of life: the respiratory vagal stimulation model of contemplative activity. *Frontiers in Human Neuroscience*. 10.3389/fnhum.2018.00397 30356789 10.3389/fnhum.2018.00397 PMC6189422

[CR23] Goshvarpour, A., & Goshvarpour, A. (2021). Asymmetry of lagged poincare plot in heart rate signals during meditation. *Journal of Traditional and Complementary Medicine,**11*(1), 16–21. 10.1016/j.jtcme.2020.01.002 33511057 10.1016/j.jtcme.2020.01.002 PMC7817711

[CR24] Grossman, P., Niemann, L., Schmidt, S., & Walach, H. (2004). Mindfulness-based stress reduction and health benefits. *Journal of Psychosomatic Research,**57*(1), 35–43. 10.1016/s0022-3999(03)00573-7 15256293 10.1016/S0022-3999(03)00573-7

[CR25] Haug, T. T., Mykletun, A., & Dahl, A. A. (2002). The prevalence of nausea in the community: Psychological, social and somatic factors. *General Hospital Psychiatry,**24*(2), 81–86. 10.1016/S0163-8343(01)00184-0 11869741 10.1016/s0163-8343(01)00184-0

[CR26] Hickey, W. S. (2010). Meditation as medicine: A critique. *CrossCurrents,**60*, 168–184. 10.1111/j.1939-3881.2010.00118.x

[CR27] Hofmann, S. G., Sawyer, A. T., Witt, A. A., & Oh, D. (2010). The effect of mindfulness-based therapy on anxiety and depression: A meta-analytic review. *Journal of Consulting and Clinical Psychology,**78*(2), 169–183. 10.1037/a0018555 20350028 10.1037/a0018555 PMC2848393

[CR28] Jevning, R., Wallace, R. K., & Beidebach, M. (1992). The physiology of meditation: A review. A wakeful hypometabolic integrated response. *Neurosci Biobehav Reviews,**16*, 415–424. 10.1016/S0149-7634(05)80210-6 10.1016/s0149-7634(05)80210-6 1528528

[CR29] Kayloni, L. O., & Emery, C. F. (2015). Mindfulness and weight loss: A systematic review. *Psychosomatic Medicine,**77*, 59–67. 10.1097/PSY.0000000000000127 25490697 10.1097/PSY.0000000000000127

[CR30] Kiyono, K., Hayano, J., Watanabe, E., & Yamamoto, Y. (2017). Heart rate variability (HRV) and sympathetic nerve activity. In S. Iwase, J. Hayano, & S. Orimo (Eds.), *Clinical assessment of the autonomic nervous system* (pp. 147–161). Berlin: Springer. 10.1007/978-4-431-56012-8_9

[CR31] Kotherová, S. (2015). Problematika experimentálního výzkumu buddhistických meditací. (problems of experimental research of Buddhist meditation). *Sociální Studia Experimenty,**12*(4), 73–93. 10.5817/SOC2015-4-73

[CR32] Kozhevnikov, M. (2019). Enhancing human cognition through vajrayana practices. *Journal of Religion and Health,**58*(3), 737–747. 10.1007/s10943-019-00776-z 30771143 10.1007/s10943-019-00776-z

[CR79] Krittanawong, C., Virk, H. U. H., Bangalore, S., Wang, Z., Johnson, K. W., Pinotti, R., Zhang, H. J., Kaplin, S., Narasimhan, B., Kitai, T., Baber, U., Halperin, J. L., & Tang, W. W. (2020). Machine learning prediction in cardiovascular diseases: A meta-analysis. *Scientific Reports, 10*(1). 10.1038/s41598-020-72685-1. 10.1038/s41598-020-72685-1 PMC7525515 32994452

[CR33] Krygier, J. R., Heathers, J. A. J., Shahrestani, S., Abbott, M., Gross, J. J., & Kemp, A. H. (2013). Mindfulness meditation, well-being, and heart rate variability: A preliminary investigation into the impact of intensive Vipassana meditation. *International Journal of Psychophysiology: Official Journal of the International Organization of Psychophysiology,**89*(3), 305–313. 10.1016/j.ijpsycho.2013.06.017 23797150 10.1016/j.ijpsycho.2013.06.017

[CR34] LaCount, L. T., Barbieri, R., Park, K., Kim, J., Brown, E. N., Kuo, B., & Napadow, V. (2011). Static and dynamic autonomic response with increasing nausea perception. *Aviation, Space, and Environmental Medicine,**82*(4), 424–433. 10.3357/asem.2932.2011 21485400 10.3357/asem.2932.2011 PMC3137518

[CR35] Lauricella, S. (2014). The ancient-turned-new concept of ‘spiritual hygiene’: An investigation of media coverage of meditation from 1979 to 2014. *Journal of Religion and Health,**55*(5), 1748–1762. 10.1007/s10943-016-0262-3 10.1007/s10943-016-0262-3 27234639

[CR36] Lee, Y. H., Shiah, Y. J., Chen, S. C., Wang, S. F., Young, M. S., & Lin, C. L. (2015). Improved emotional stability in experienced meditators with concentrative meditation based on electroencephalography and heart rate variability. *Journal of Alternative and Complementary Medicine,**21*(1), 31–39. 10.1089/acm.2013.0465 25354314 10.1089/acm.2013.0465

[CR37] Lehrer, P., Sasaki, Y., & Saito, Y. (1999). Zazen and cardiac variability. *Psychosomatic Medicine,**61*, 812–821. 10.1016/j.tics.2008.01.005 10593633 10.1097/00006842-199911000-00014

[CR38] Levine, G. N., Lange, R. A., Bairey-Merz, C. N., Davidson, R. J., Jamerson, K., Mehta, P. K., Michos, E. D., Norris, K., Ray, I. B., Saban, K. L., Shah, T., Stein, R., & Smith, S. C., Jr. (2017). American heart association council on clinical cardiology; council on cardiovascular and stroke nursing; and council on hypertension. Meditation and cardiovascular risk reduction: A scientific statement from the American heart association. *Journal of the American Heart Association,**6*(10), e002218. 10.1161/JAHA.117.002218 28963100 10.1161/JAHA.117.002218 PMC5721815

[CR39] Lewek, J., Wranicz, J. K., Guzik, P., Chudzik, M., Ruta, J., & Cygankiewicz, I. (2009). Clinical and electrocardiographic covariates of deceleration capacity in patients with ST-segment elevation myocardial infarction. *Cardiology Journal,**16*(6), 528–534. 19950089

[CR40] Lindahl, J. R., Fisher, N. E., Cooper, D. J., Rosen, R. K., & Britton, W. B. (2017). The varieties of contemplative experience: A mixed-methods study of meditation-related challenges in Western Buddhists. *PLoS ONE,**12*(5), e0176239. 10.1371/journal.pone.0176239 28542181 10.1371/journal.pone.0176239 PMC5443484

[CR41] Lomas, T., Cartwright, T., Edginton, T., & Ridge, D. (2015). A qualitative analysis of experiential challenges associated with meditation practice. *Mindfulness,**6*, 848–860. 10.1007/s12671-014-0329-8

[CR42] Lustyk, MK., Chawla, N., Nolan, RS. & Marlatt, GA. (2009). Mindfulness meditation research: issues of participant screening, safety procedures, and researcher training. *Advances in Mind-Body Medicine, Spring, 24*(1), 20–30. https://pubmed.ncbi.nlm.nih.gov/20671334/20671334

[CR43] Lutkajtis, A. (2019a). The dark side of dharma: why have adverse effects of meditation been ignored in contemporary Western secular contexts? *Journal for the Academic Study of Religion,**31*(2), 192–217. 10.1558/jasr.37053

[CR44] Lutkajtis, A. (2019b). ’The answer to all your problems?’ The overly positive presentation of meditation in the media. *Journal for the Academic Study of Religion,**32*(1), 49–71. 10.1558/jasr.37863

[CR45] Lutz, A., Slagter, H. A., Dunne, J. D., & Davidson, R. J. (2008). Attention regulation and monitoring in meditation. *Trends in Cognitive Sciences,**12*, 163–169. 10.1016/j.tics.2008.01.005 18329323 10.1016/j.tics.2008.01.005 PMC2693206

[CR46] Malik, M. (1996). Heart rate variability: Standards of measurement, physiological interpretation, and clinical use: Task force of the European Society of cardiology and the North American Society for pacing and electrophysiology. *Annals of Noninvasive Electrocardiology,**1*(2), 151–181. 10.1111/j.1542-474X.1996.tb00275.x

[CR47] McCorry, L. K. (2007). Physiology of the autonomic nervous system. *American Journal of Pharmaceutical Education,**71*(4), 78. 10.5688/aj710478 17786266 10.5688/aj710478 PMC1959222

[CR49] Muth, E. R., Stern, R. M., Thayer, J. F., & Koch, K. L. (1996). Assessment of the multiple dimensions of nausea: The nausea profile (NP). *Journal of Psychosomatic Research,**40*(5), 511–520. 10.1016/0022-3999(95)00638-9 8803860 10.1016/0022-3999(95)00638-9

[CR50] Ñāṇamoli, B. (1998). *Mindfulness of breathing (anapanasati): buddhist texts from the pali canon and extracts from the pali commentaries*. Kandy: Buddhist Publication Society.

[CR51] Nilsson, H., & Kazemi, A. (2016). From Buddhist sati to Western mindfulness practice: A contextual analysis. *Journal of Religion & Spirituality in Social Work: Social Thought,**35*, 7–23. 10.1080/15426432.2015.1067582

[CR52] Olex, S., Newberg, A., & Figueredo, V. M. (2013). Meditation: Should a cardiologist care? *International Journal of Cardiology,**168*(3), 1805–1810. 10.1016/j.ijcard.2013.06.086 23890919 10.1016/j.ijcard.2013.06.086

[CR53] Ospina, MB., Bond, TK., Karkhaneh, M., Tjosvold, L., Vandermeer B., Liang, Y., Bialy, L., Hooton, N., Buscemi, N., Dryden, DM. & Klassen, TP. (2007). Meditation practices for health: State of the research. Rockville: Evidence report/technology assessment. agency for healthcare research and quality. *Evidence Report/Technology Assessment (Full Rep)*, Jun, *155*, 1–263. https://pubmed.ncbi.nlm.nih.gov/17764203/PMC4780968 17764203

[CR54] Pan, Q., Zhou, G., Wang, R., et al. (2016). Do the deceleration/acceleration capacities of heart rate reflect cardiac sympathetic or vagal activity? A model study. *Medical & Biological Engineering & Computing,**54*(12), 1921–1933. 10.1007/s11517-016-1486-9 27059998 10.1007/s11517-016-1486-9

[CR55] Peng, C. K., Henry, I. C., Mietus, J. E., Hausdorff, J. M., Khalsa, G., Benson, H., & Goldberger, A. L. (2004). Heart rate dynamics during three forms of meditation. *International Journal of Cardiology,**95*(1), 19–27. 10.1016/j.ijcard.2003.02.006 15159033 10.1016/j.ijcard.2003.02.006

[CR56] Raffone, A., Marzetti, L., Del Gratta, C., Perrucci, M. G., Romani, G. L., & Pizzella, V. (2019). Toward a brain theory of meditation. In N. Srinivasan (Ed.), *Progress in brain research* (Vol. 244, pp. 207–232). Amsterdam: Elsevier. 10.1016/bs.pbr.2018.10.028 10.1016/bs.pbr.2018.10.028 30732838

[CR57] Rubia, K. (2009). The neurobiology of meditation and its clinical effectiveness in psychiatric disorders. *Biological Psychology,**82*(1), 1–11. 10.1016/j.biopsycho.2009.04.003 19393712 10.1016/j.biopsycho.2009.04.003

[CR58] Rusch, H. L., Rosario, M., Levison, L. M., Olivera, A., Livingston, W. S., Wu, T., & Gill, J. M. (2019). The effect of mindfulness meditation on sleep quality: A systematic review and meta-analysis of randomized controlled trials. *Annals of the New York Academy of Sciences,**1445*(1), 5–16. 10.1111/nyas.13996 30575050 10.1111/nyas.13996 PMC6557693

[CR59] Saeed, S. A., Cunningham, K., & Bloch, R. M. (2019). Depression and anxiety disorders: Benefits of exercise, yoga, and meditation. *American Family Physician*, *99*(10), 620–627. https://pubmed.ncbi.nlm.nih.gov/31083878/31083878

[CR60] Sanger, G. J., & Andrews, P. L. R. (2023). Review article: An analysis of the pharmacological rationale for selecting drugs to inhibit vomiting or increase gastric emptying during treatment of gastroparesis. *Alimentary Pharmacology & Therapeutics,**57*(9), 962–978. 10.1111/apt.17466 36919196 10.1111/apt.17466

[CR80] Schnaubelt, S., Hammer, A., Koller, L., Niederdöckl, J., Kazem, N., Spiel, A., Niessner, A., & Sulzgruber, P. (2019). Meditation and Cardiovascular Health: What is the Link? *European Cardiology, 14*(3), 161–164. 10.15420/ecr.2019.21.2 10.15420/ecr.2019.21.2 PMC6950207 31933684

[CR61] Sclocco, R., Kim, J., Garcia, R. G., Sheehan, J. D., Beissner, F., Bianchi, A. M., & Napadow, V. (2016). Brain circuitry supporting multi-organ autonomic outflow in response to nausea. *Cerebral Cortex,**26*(2), 485–497. 10.1093/cercor/bhu172 25115821 10.1093/cercor/bhu172 PMC4712791

[CR62] Scott-Sheldon, L. A., Gathright, E. C., Donahue, M. L., Balletto, B., Feulner, M. M., DeCosta, J., & Salmoirago-Blotcher, E. (2020). Mindfulness-based interventions for adults with cardiovascular disease: a systematic review and meta-analysis. *Annals of Behavioral Medicine,**54*(1), 67–73. 10.1093/abm/kaz020 31167026 10.1093/abm/kaz020 PMC6922300

[CR78] Shapiro, D. H. (1992). A preliminary study of long term meditators: Goals, effects, religious orientation, cognitions. *The Journal of Transpersonal Psychology,**24*(1), 23–39.

[CR64] Shr-Da, W., & Pei-Chen, L. (2008). Inward-attention meditation increases parasympathetic activity: A study based on heart rate variability. *Biomedical Research,**29*, 245–250. 10.2220/biomedres.29.245 18997439 10.2220/biomedres.29.245

[CR66] Tang, Y. Y., Ma, Y., Wang, J., Fan, Y., Feng, H., Wang, J., Feng, A., Lu, Q., Hu, B., Lin, Y., Li, J., Zhang, Y., Wang, Y., Zhou, L., & Fan, M. (2009). Central and autonomic nervous system interaction is altered by short-term meditation. *Proceedings of the National Academy of Sciences of the United States of America,**106*, 8865–8870. 10.1073/pnas.0904031106 19451642 10.1073/pnas.0904031106 PMC2690030

[CR67] Tarbell, S. E., Olufs, E. L., Fischer, P. R., Chelimsky, G., Numan, M. T., Medow, M., Abdallah, H., Ahrens, S., Boris, J. R., Butler, I. J., Chelimsky, T. C., Coleby, C., Fortunato, J. E., Gavin, R., Gilden, J., Gonik, R., Klaas, K., Marsillio, L., Marriott, E., & Weese-Mayer, D. E. (2023). Assessment of comorbid symptoms in pediatric autonomic dysfunction. *Clinical Autonomic Research,**33*(6), 843–858. 10.1007/s10286-023-00984-4 37733160 10.1007/s10286-023-00984-4

[CR69] Taylor, G. B., Vasquez, T. S., Kastrinos, A., Fisher, C. L., Puig, A., & Bylund, C. L. (2022). The adverse effects of meditation-interventions and mind-body practices: A systematic review. *Mindfulness,**13*(8), 1839–1856. 10.1007/s12671-022-01915-6

[CR71] Van Dijk, J. G., Ghariq, M., Kerkhof, F. I., Reijntjes, R., Van Houwelingen, M. J., Van Rossum, I. A., & Benditt, D. G. (2020). Novel methods for quantification of vasodepression and cardioinhibition during tilt-induced vasovagal syncope. *Circulation Research,**127*(5), e126–e138. 10.1161/circresaha.120.316662 32460687 10.1161/CIRCRESAHA.120.316662

[CR72] Wang, Y. P., Kuo, T. B. J., Li, J. Y., Lai, C. T., & Yang, C. C. H. (2016). The relationships between heart rate deceleration capacity and spectral indices of heart rate variability during different breathing frequencies. *European Journal of Applied Physiology,**116*, 1281–1287. 10.1007/s00421-016-3332-z 26832134 10.1007/s00421-016-3332-z

[CR73] Won, E., & Kim, Y.-K. (2016). Stress, the autonomic nervous system, and the immune-kynurenine pathway in the etiology of depression. *Current Neuropharmacology,**14*(7), 665–673. 10.2174/1570159x14666151208113006 27640517 10.2174/1570159X14666151208113006 PMC5050399

[CR74] Wong, S. Y. S., Chan, J. Y. C., Zhang, D., Lee, E. K. P., & Tsoi, K. K. F. (2018). The safety of mindfulness-based interventions: A systematic review of randomized controlled trials. *Mindfulness,**9*(5), 1344–1357. 10.1007/s12671-018-0897-0

[CR75] Young, J.D.-E., & Taylor, E. (1998). Meditation as a voluntary hypometabolic state of biological estivation. *Physiology (bethesda, Md.),**13*(3), 149–153. 10.1152/physiologyonline.1998.13.3.149 10.1152/physiologyonline.1998.13.3.149 11390779

[CR82] Younge, J. O., Gotink, R. A., Baena, C. P., Roos-Hesselink, J. W., & Hunink, M. G. (2015). Mind-body practices for patients with cardiac disease: a systematic review and meta-analysis. *European Journal of Preventive Cardiology,**22*(11), 1385–1398. 10.1177/2047487314549927 25227551 10.1177/2047487314549927

[CR77] Zhang, F., Zhang, Y., Jiang, N., Zhai, Q., Hu, J., & Feng, J. (2021). Influence of mindfulness and relaxation on treatment of essential hypertension: Meta-analysis. *Journal of Healthcare Engineering,**2021*, 1–7. 10.1155/2021/2272469 10.1155/2021/2272469 PMC8664515 34900178

